# Nutritional advantages of sous‐vide cooking compared to boiling on cereals and legumes: Determination of ashes and metals content in ready‐to‐eat products

**DOI:** 10.1002/fsn3.469

**Published:** 2017-03-01

**Authors:** Mariangela Rondanelli, Maria Daglia, Silvia Meneghini, Arianna Di Lorenzo, Gabriella Peroni, Milena Anna Faliva, Simone Perna

**Affiliations:** ^1^ Department of Public Health, Experimental and Forensic MedicineSchool of Medicine, Endocrinology and Nutrition UnitUniversity of PaviaAzienda di Servizi alla Persona di PaviaPaviaItaly; ^2^ Department of Drug Sciences, Medicinal Chemistry and Pharmaceutical Technology SectionUniversity of PaviaPaviaItaly

**Keywords:** ashes, cereals, legumes, minerals, sous vide, traditional cooking

## Abstract

In order to guarantee the highest quality of ready‐to‐eat cereals and legumes, two different cooking methods have been applied: traditional cooking and sous‐vide. Ashes and metals content (magnesium, potassium, iron, zinc, and copper) has been determined and compared in 50 samples of red lentils, peas, Borlotti beans, pearl barley, and cereals soup. All the samples cooked with sous‐vide showed a significant increase in the content of minerals with the exception of potassium in cereal soup, iron in Borlotti beans, and magnesium in pearl barley. Ash content increased in legumes and in cereal soup cooked with sous‐vide method. The higher different ashes concentration between total samples cooked with traditional cooking and with sous‐vide was registered in zinc (+862 mg), iron (+314 mg), potassium (+109 mg), and copper (+95 mg). Sous‐vide is preferred as it provides products with a higher concentration of metals compared to the ones cooked with traditional cooking.

## 1 Introduction

Health is widely regarded as the essential benefit/resource and it is defined as a state of physical, mental, and social well‐being achieved by a balanced diet and regular physical activity (WHO, 1948). As the human body needs the right amount of nutrients and bioactive compounds, that is nutraceuticals, a varied diet is required to prevent nutritional and metabolic imbalances (Kalt, 2005; Leong et al., 2013; Nabavi, Russo, Daglia, & Nabavi, 2015).

Nowadays, it is important to know not only the type of food that should be consumed, but also the technological and conservation treatments which are preferable in order to assume food with high nutritional values (Li et al., 2016; Ramos dos Reis et al., 2015) and hygienically processed. Moreover, growing number of health‐conscious consumers are focusing their attention on food choices even if they lack of time to prepare fresh vegetable dishes and opt for ready‐to‐eat replacements.

Cooking is the main treatment that foods undergo, which has the aim of prolonging storage time, providing an inviting aspect and color, destroying microorganisms and deactivating anti‐nutrients or toxic substances naturally present in raw product and of improving digestibility and sensory characteristics, such as flavor and aroma (Iborra‐Bernad, Tárrega, García‐Segovia, & Martínez‐Monzó, 2014a). However, the cooking process has also negative effects as the reduction of nutritive values caused by the degradation of thermo‐labile vitamins (especially vitamin C and B group vitamins), the destruction of some essential aminoacids, and the leakage of mineral salts and vitamins in cooking water. For example, different studies suggest that heat causes structural changes in meat, such as the destruction of cell membranes and denaturation of proteins as well as the loss of physical properties (color, texture) (García‐Segovia, Andrés‐Bello, & Martínez‐Monzó, 2007; Tornberg, 2005). Moreover, cooking can cause also the formation of toxic substances, harmful to the body, such as the ones resulting from charring by grilling and frying. Therefore, the choice of cooking method is far from trivial/ordinary.

Among foods, vegetables and fruits, whose dietary intake should be at least five portions each day, play a key role in human diet thanks to their components such as vitamins, minerals, fiber, and phytochemicals components (Rekhy & McConchie, 2014).

As matter of facts, as showed in the study by Natella, Belelli, Ramberti, & Scaccini (2010); the comparison of the effects of different cooking methods on the seven vegetables analyzed in this study indicates that microwave and pressure cooking are less detrimental than boiling to the phenolics content of vegetables. In addition The preservation of the antioxidant capacity in vegetables depends on the kind of vegetable and/or cooking procedure (Natella et al., 2010).

Some of them are eaten raw, while others are cooked before their consumption. In the case of cereals and legumes which are sources of nutrients such as carbohydrates, proteins, vitamins and mineral salts, and bioactive compounds (fiber), the cooking process is necessary to allow their consumption. To cook cereals is a way to soften the cellulose and then it enables the consumers to chew foods, whereas cooking legumes provides the inactivation of anti‐nutritional substances that hinder the digestion of nutrients and could have negative effects on the health of the consumer.

Generally, the most common methods to cook vegetables are boiling, named also traditional cooking, and steaming. Both of them require high temperature (around 100°C) and the presence of oxygen which can lead to a decrease in nutritional substances and can influence the activity and bioavailability of active compounds. Therefore, under sous‐vide cooking is a possible strategy to avoid loss of nutrients, minerals, and vitamins. Indeed, different studies suggest that the loss of molecules in vegetables, like anthocyanin, ascorbic acid, and polyphenols were lower applying sous‐vide conditions (Baardseth, Bjerke, Martinsen, & Skrede, 2010; Iborra‐Bernad, García, & Martínez‐Monzó, 2015; Renna, Gonnella, Giannino, & Santamaria, 2014). As a result, thus this food presents a higher antioxidant activity compared to the one cooked with traditional cooking. Sous‐vide and cook‐vide are the most important under sous‐vide treatments which are characterized by the use of temperature below 100°C and absence of the oxygen species. This paper is focused on sous‐vide treatment in which foods were put inside a heat‐stable sous‐vide pouches and slowly cooked under controlled conditions of temperature (around 90°C) and time (Baldwin, 2012). Sous‐vide provides different advantages in cooking not only associated to nutritional values but also to the sensorial satisfaction of consumers (Iborra‐Bernad, Tárrega, García‐Segovia, & Martínez‐Monzó, 2014b; Renna et al., 2014). Indeed, vegetables were tastier and more aromatic than the ones cooked via boiling method. Sous‐vide packaging prevents the direct contact between food and oxygen reducing the oxidation of pigments such as chlorophyll and carotenoids. Moreover, it improves food security and it allows longer storage of the prepared food.

Finally, sous‐vide offers greater advantages among cooking methods to which vegetables can be exposed including the conservation of nutritional values, the quality and safety of the food, and a more attractive flavor (García‐Segovia et al., 2007 Iborra‐Bernad et al., 2015).

Given this background, the aim of the present study is to evaluate the influence of the cooking methods (traditional boiling and sous‐vide) on ashes and metals content, which are, respectively, measure of the total amount of minerals and measure of the amount of specific inorganic components, such as Mg, Fe, K, and Zn, present in ready‐to‐eat cereals and legumes (red lentils, peas, Borlotti beans, pearl barley, and cereal soup). Determination of the ash and metal content is important for parameters as quality, microbiological stability, and nutritional values of foods. Indeed, mineral salts are useful in tissues and are essential factors for the biological functions as they are involved in structural and regulation activities.

## 2 Materials and Methods

### 2.1 Materials and cooking methods

Ashes and metals content (magnesium, potassium, iron, zinc, and copper) has been determined and compared in 50 samples of red lentils, peas, Borlotti beans, pearl barley, and cereals soup.

We used for the analysis were prepared from So.Vite S.p.A. company (Giussago, PV, Italy) and cooked with two different methods: the traditional cooking (boiling water at atmospheric pressure) and the sous‐vide treatment, as shown in Table 1.

**Table 1:** Cooking methods

	Traditional cooking	Sous‐vide treatment
Cooking temperature	Product brought to the boil	65°C Test1–74°C Test2
Cooking time	1 hr	10 hr Test1 4 hr Test 2
Size	1 kg of products in 4 kg in water, and after cooking, in boxes of 2 kg and of 250 g	Boxes of 2 kg Boxes of 250 g

One portions of cereal soup has been prepared with: bacon (1 g), dried red lentils (6 g), fresh peas (6 g), fresh beans (6 g), barley (3 g), spelled (4 g), carrots (5 g), onions (5 g), potatoes (3 g), and extra‐virgin olive oil (3 ml).

### 2.2 Determination of ashes content

The ashes content was determined in each sample according to the method reported in “Metodi di Analisi utilizzati per il Controllo Chimico degli Alimenti” (Baldini et al., 1996). The method involves the insertion of an aliquot of the sample in oxidizing medium at 550 ± 10°C until complete combustion of the organic substance and achievement of a constant mass. The procedure involves the weighing of the solid samples (from 2 g to 10 g) in a calibrated capsule according to the expected ashes yield. The capsules were placed on the heating pad and carefully warmed in order to prevent the leakage of material particles. Afterwards the residues were placed inside the oven previously heated to 550 ± 10°C until complete combustion of all the carbon particles contained in them. At the end of the incineration, the capsules were removed from the oven and cooled in the desiccator until room temperature. The residues were quickly weighed with a precision of 0.0001 g. The three steps (heating, cooling, and weighing) were repeated until the attainment of a constant mass. Ashes sample content, expressed as percentage, was calculated as follows:

$$\text{Ashes}\;\left(\text{mg}/100g\right)=\left(m_{1}/m_{0}\right)\times 100\times 1000,$$

where *m*_0_ = mass, expressed in grams, of aliquot of the sample analyzed; *m*_1_ = mass, expressed in grams, of the residue.

### 2.3 Determination of metals content

As for the previous determination, metals were revealed according to the method reported in “Metodi di Analisi utilizzati per il Controllo Chimico degli Alimenti” (Baldini et al., 1996). The mineralization process requires 1 g of each homogenized sample to which was added 25 ml of nitric acid 65% and 1 ml of sulfuric acid 96%. Afterwards they were heated gradually with a reflux system until the preparations became pales. The solutions were cooled and a little volume of them were concentrated on a hotplate. The residues were moved into a volumetric flask (25 ml) and filled to the mark with bidistilled water. The mineralization process was performed also for the blank, omitting the portion of the sample to be analyzed. The obtained solutions were analyzed and magnesium (Mg), potassium (K), iron (Fe), copper (Cu), and zinc (Zn) content was determined using atomic absorption spectroscopy (AAS) and expressed as mg/100 g of product.

### 2.4 Statistical analysis

SPSS statistical software (version 21.0, SPSS Inc., Chicago, IL) was used to perform paired *t*‐test to compare the means of the two samples of related data between sous‐vide minus traditional cooking.

## 3 Results

**Figure 1:**
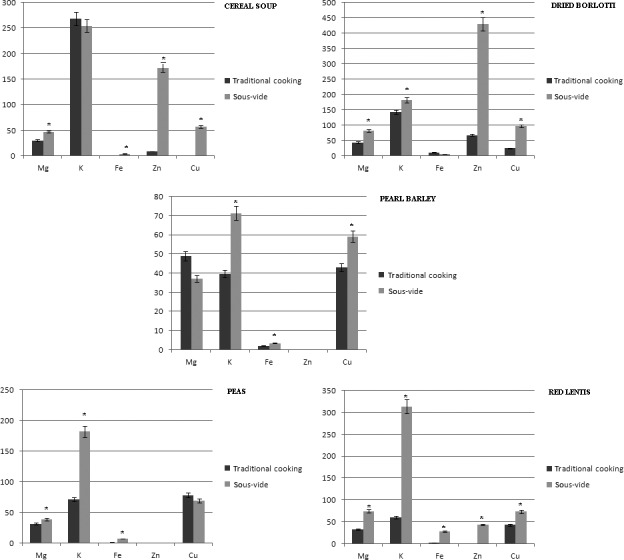
Measure of the total amount of minerals expressed in mg/100 g of cooked product in all six samples cooked with traditional cooking (T) and sous‐vide (SV)

Table 1 showed the cooking methods in comparison. As regards cereal soup, the content of all minerals, with the exception of potassium, it is higher in the cereal soup cooked sous‐vide than conventional cooking, as shown in Table 2 and in Figure 1.

**Table 2:** Measure of the total amount of minerals expressed in mg/100 g of cooked product of cereal soup cooked with traditional cooking (T) and sous‐vide (SV)

Minerals	Traditional cooking mean ± *SD*	Sous‐vide mean ± *SD*	Δ Change	CI (95%) low	CI (95%) high
Magnesium (mg)	30.22 ± 1.01	47.12 ± .95	16.90	14.68	19.12
Potassium (mg)	268.5 ± 3.05	254.75 ± 3.71	−13.75	−21.45	−6.05
Iron (mg)	1.22 ± 0.01	4.27 ± 0.09	3.05	2.9	3.2
Zinc (mg)	8.85 ± 0.17	172.42 ± 3.22	163.57	158.4	168.74
Copper (mg)	0.00 ± 0.00	57.12 ± 1.23	57.12	55.15	59.09

As for the beans, the content of all minerals, with the exception of the iron, is higher when the beans are cooked under sous‐vide with respect to traditional cooking, as shown in Table 3 and in Figure 1.

**Table 3:** Measure of the total amount of minerals expressed in mg/100 g of cooked product of Borlotti beans cooked with traditional cooking (T) and sous‐vide (SV)

Minerals	Traditional cooking mean ± *SD*	Sous‐vide mean ± *SD*	Δ Change	CI low	CI high
Magnesium (mg)	43.92 ± 1.02	82.82 ± 2.07	38.90	35.2	42.6
Potassium (mg)	143.25 ± 3.92	182.35 ± 2.56	39.10	31.6	46.6
Iron (mg)	11.72 ± 0.25	5.92 ± 0.23	−5.80	−6.34	−5.26
Zinc (mg)	68.10 ± 1.95	430.25 ± 8.10	362.15	348.79	375.51
Copper (mg)	25.10 ± 0.64	97.20 ± 2.04	72.10	68.67	75.53

Regarding pearl barley, the content of all minerals, with the exception of magnesium, it is higher in sous‐vide cooked pearled barley compared with traditional cooking, as shown in Table 4 and in Figure 1.

**Table 4:** Measure of the total amount of minerals expressed in mg/100 g of cooked product of pearl barley cooked with traditional cooking (T) and sous‐vide (SV)

Minerals	Traditional cooking mean ± *SD*	Sous‐vide mean ± *SD*	Δ Change	CI 95% low	CI 95% high
Magnesium (mg)	49.07 ± 0.88	37.12 ± 0.97	−11.95	−14.05	−9.85
Potassium (mg)	39.82 ± 0.86	71.47 ± 1.82	31.65	28.42	34.88
Iron (mg)	2.10 ± 0.04	3.60 ± 0.12	1.50	1.3	1.7
Zinc (mg)	–		–	–	–
Copper (mg)	43.07 ± 0.97	59.20 ± 1.26	16.13	13.58	18.68

As regards peas, the content of all minerals, with the exception of copper, is higher in peas cooked under sous‐vide with respect to traditional cooking, as shown in Table 5 and in Figure 1.

**Table 5:** Measure of the total amount of minerals expressed in mg/100 g of cooked product of peas cooked with traditional cooking (T) and sous‐vide (SV)

Minerals	Traditional cooking mean ± *SD*	Sous‐vide mean ± *SD*	Δ Change	CI 95% low	CI 95% high
Magnesium (mg)	31.52 ± 0.84	39.00 ± 0.91	7.48	5.49	9.47
Potassium (mg)	71.42 ± 0.71	182.10 ± 2.64	110.68	106.3	115.06
Iron (mg)	1.37 ± 0.03	7.22 ± 0.14	5.85	5.62	6.08
Zinc (mg)	–	–	–	–	–
Copper (mg)	78.45 ± 1.67	69.17 ± 1.58	−9.28	−12.97	−5.59

As for red lentils, the content of all minerals, is higher in red lentils cooked under sous‐vide with respect to traditional cooking, as shown in Table 6 and in Figure 1.

**Table 6:** Measure of the total amount of minerals expressed in mg/100 g of cooked product of red lentis cooked with traditional cooking (T) and sous‐vide (SV)

Minerals	Traditional cooking mean ± *SD*	Sous‐vide mean ± *SD*	Δ Change	CI 95% Low	CI 95% High
Magnesium (mg)	33.07 ± 0.83	74.80 ± 1.62	41.73	38.81	44.65
Potassium (mg)	60.32 ± 0.32	313.50 ± 6.10	253.18	243.39	262.97
Iron (mg)	2.40 ± 0.04	29.10 ± 0.88	26.70	25.29	28.11
Zinc (mg)	0.00 ± 0.00	43.65 ± 0.57	43.65	42.74	44.56
Copper (mg)	43.52 ± 1.12	73.85 ± 1.89	30.33	26.81	33.85

^a^Δ Change: amount of minerals expressed in mg preserved by sous‐vide cooking (mean of minerals by sous‐vide cooking minus mean of minerals by traditional cooking); ^b^CI 95%: confidence interval of mean change.

**Figure 2:**
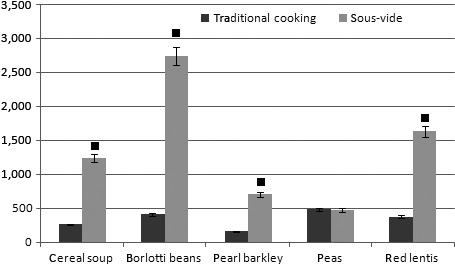
Measure of the total amount of Ashes expressed in mg/100 g of cooked product in all six samples cooked with traditional cooking (T) and sous‐vide (SV)

Considering the ash content, as shown in Table 7 and in Figure 2, it increased in all legumes and in cereal soup cooked with sous‐vide method compared to traditional cooking. In particular, as shown in Figure 2, the higher different ashes concentration between total samples cooked with traditional cooking and with sous‐vide was registered in, respectively, in zinc (+862 mg), in iron (+314 mg), in potassium (+109.42 mg), in copper (+95.64 mg), and magnesium.

**Table 7:** Measure of the amount of specific inorganic components (ashes) expressed in mg/100 g of cooked product in all six samples cooked with traditional cooking (T) and sous‐vide (SV)

Minerals	Traditional cooking mean ± *SD*	Sous‐vide mean ± *SD*	Δ Change	CI 95% Low	CI 95% High
Cereal soup	260 ± 13	1240 ± 21	980.00	−1022.89	−937.10
Borlotti beans	412 ± 12	2737 ± 82	2321.66	−2532.47	−2110.86
Pearl barley	157 ± 4	699 ± 21	541.33	−588.59	−494.07
Peas	486 ± 15	473 ± 14	−13.00	−19.95	45.95
Red lentis	374 ± 11	1629 ± 48	1137.66	−1634.48	−640.84

Traditional cooking\sous‐vide: mean of minerals expressed in mg/100 g after cooking; Δ Change: amount of minerals expressed in mg preserved by sous‐vide cooking (mean of minerals by sous‐vide cooking minus mean of minerals by traditional cooking); CI 95%: confidence interval of mean change.

Copper is completely absent in the cereal soup cooked with traditional boiling, but is always present in legumes and cereal soup sous‐vide cooked (except for peas).

Zinc is completely absent in pearl barley, red lentils, and peas cooked with traditional brewing. In sous‐vide cooking zinc is absent after cooking peas and pearl barley and the content was higher in cereal soup, kidney beans, and red lentils.

The magnesium content is always higher in all legumes and cereal soup cooked with sous‐vide method compared with traditional cooking, except that in pearl barley. Moreover, the concentration of Mg in all samples is higher in sous‐vide cooked ones with the exception of pearl barley (37 mg/100 g of cooked product with sous‐vide method and 49 mg/100 g of cooked product with boiling; ∆ = −24.35%).

The potassium content is always higher in all legumes cooked with sous‐vide method than conventional cooking, whereas in the cereal soup the potassium content is higher when cooked with traditional method.

We highlight that the iron content was higher in all legumes, with the exception of Borlotti beans, and cereal soup cooked with sous‐vide method.

According to our results reported in Figure 1, All the samples cooked with sous‐vide showed an increase in the content of minerals with the exception of potassium in cereal soup (−6.05 mg), iron in Borlotti beans (−6.34 mg), and magnesium in pearl barley (−14.05 mg).

**Figure 3:**
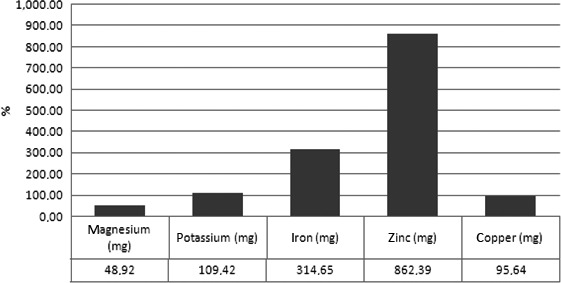
Minerals preserved in addition (on average in “mg” in all foods considered) with sous‐vide cooking over traditional cooking

**Figure 4:**
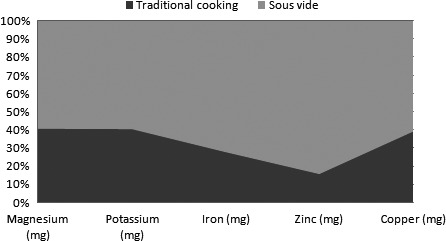
Minerals preserved in addition (on average in “percentage” in all foods considered) with sous‐vide cooking over traditional cooking)

**Figure 5:**
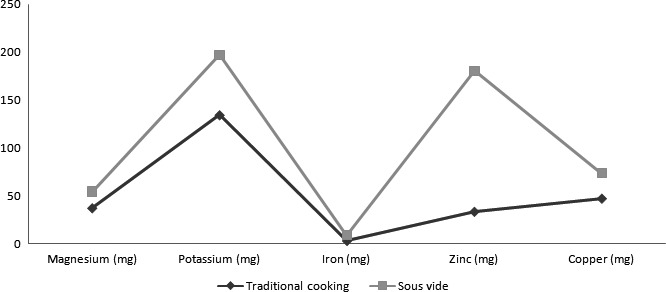
Minerals preserved in addition (on average in “mg” in all foods considered) with sous‐vide cooking over traditional cooking

Figures 3, 4, 5 show minerals preserved in addition (on average in “mg” in all foods considered) with sous‐vide cooking over traditional cooking).

## 4 Discussion

This paper, for the first time in the literature, has evaluated the content of ash and minerals (magnesium, potassium, iron, zinc, and copper) in legumes (red lentils, peas, Borlotti beans) and cereals (pearl barley and cereal soup) after cooking with sous‐vide methods and having compared the mineral content of legumes and cereals cooked under sous‐vide or cooked with traditional method (boiling). The results of this study demonstrate how the sous‐vide cooking allows to better preserve the content of minerals than the boiling: in fact all the samples cooked with sous‐vide showed an increase in the content of minerals with the exception of potassium in cereal soup (−6.05 mg), iron in Borlotti beans (−6.34 mg), and magnesium in pearl barley (−14.05 mg).

Moreover, regarding the ash content, it increased in all legumes and in cereal soup cooked with sous‐vide method compared to traditional cooking. In particular, the higher different ashes concentration between total samples cooked with traditional cooking and with sous‐vide was registered, respectively, in zinc (+862 mg), in iron (+314 mg), in potassium (+109 mg), in copper (+95 mg), and magnesium.

To date there have been no studies that have evaluated the mineral content in sous‐vide cooked foods, while the literature agrees in defining that by boiling there is a loss of minerals comprised between 20% and 40% (Engler‐Stringer, 2010; Kimura & Itokawa, 1990; Meiners et al., 1976; Severi et al., 1998).

Conversely, sous‐vide is an emerging cooking technique that is reputed to provide superior quality owing to the low amount of oxygen inside the pack (Tansey, Gormley, & Butler, 2010; Martinez‐Hernandez et al. 2013) and because of its capacity to retain more of the nutritive value and sensory characteristics of the vegetables compared with other conventional methods (Creed, 1995; Petersen, 1993; Stea, Johansson, Jägerstad, & Frølich, 2007; Werlein, 1998).

Using a heat treatment to guarantee a longer shelf life of, say, 21 days will unnecessarily degrade the sensory and nutritional qualities of the product if it would normally be used after 10 days of storage. Food quality as perceived by the consumer is not something which can be broken down into neat components labeled as scores for “taste,” “texture,” “odor,” or measured quantities such as “acidity” or “fat content,” etc. Claims for sous‐vide foods often involve emotional and psychological responses linked to many factors associated with the eating environment (Creed, 1998).

Our results, although the minerals have never been evaluated in sous‐vide cooked foods, consistent with previous literature that demonstrates that the sous‐vide cooking has nutritional and healthy benefits, preserving the vitamin content and antioxidant capacity (Creed, 1995; Patras, Brunton, & Butler, 2010; Petersen, 1993; Stea et al., 2007).

The main limitation of this study is the fact that it was not assessed the content of the mineral in raw foods in order to compare the content of minerals in the raw food and then in cooked foods with the two cooking methods (boiled or sous‐vide). Further studies are therefore needed in order to evaluate the mineral content in raw food and then cooked food with different cooking methods in order to advise the consumer on what cooking methods are the best to preserve the most of the minerals.

## 5 Conclusions

In conclusions, as a whole, these results suggest that minerals salts present in legumes and cereals were dispersed in cooking water when prepared with the traditional method, whereas sous‐vide treatment keep them also in the cooked food done for the consume. Overall, we can assume that vegetables samples cooked with sous‐vide treatment exhibit higher concentrations of minerals compared to the same ones cooked with the traditional method. Legumes and cereals sous‐vide cooking is the method which offers greatest results keeping nutritional values in addition to intense flavors and hygienically safe foods.

## Conflict of Interest

The authors declare no conflict of interest.
